# Fibroblast Growth Factors in Lung Development and Regeneration: Mechanisms and Therapeutic Potential

**DOI:** 10.3390/cells14161256

**Published:** 2025-08-14

**Authors:** Karolina Baran, Kamila Skrzynska, Aleksandra A. Czyrek, Adrianna Wittek, Daniel Krowarsch, Anna Szlachcic, Malgorzata Zakrzewska, Julia Chudzian

**Affiliations:** 1 Department of Protein Engineering, Faculty of Biotechnology, University of Wroclaw, 50-383 Wroclaw, Poland; karolina.baran@uwr.edu.pl (K.B.); kamila.skrzynska@uwr.edu.pl (K.S.); adrianna.wittek@gmail.com (A.W.); daniel.krowarsch@uwr.edu.pl (D.K.); anna.szlachcic@gmail.com (A.S.); 2 Department of Biology, Faculty of Medicine, Masaryk University, 62500 Brno, Czech Republic; aleksandra.anna.czyrek@med.muni.cz; 3 International Clinical Research Center, St. Anne’s University Hospital, 65691 Brno, Czech Republic

**Keywords:** fibroblast growth factors, FGFR signaling, lung development, lung regeneration

## Abstract

Fibroblast growth factors (FGFs) play a key role in lung development by mediating complex interactions between epithelial and mesenchymal cells, which are central to processes such as branching morphogenesis, epithelial differentiation, and alveolarization. The findings regarding this interplay highlight the complexity of FGF signaling, as different FGFs contribute to various aspects of lung formation and maturation. Understanding the role of FGF proteins in shaping the lung is crucial for gaining insight into the biology of its development. Furthermore, FGFs orchestrate complex signaling pathways that regulate lung regeneration in adulthood. Therapeutic strategies targeting FGF-dependent pathways appear promising for repairing and improving lung function in diverse pulmonary diseases. In this review, we describe the current perception of the role of FGF proteins in lung development and regeneration, together with an overview of emerging therapeutic strategies aiming at FGF signaling in lung-related disorders.

## 1. An Overview of the Morphological Development of Lungs

**Figure 1:**
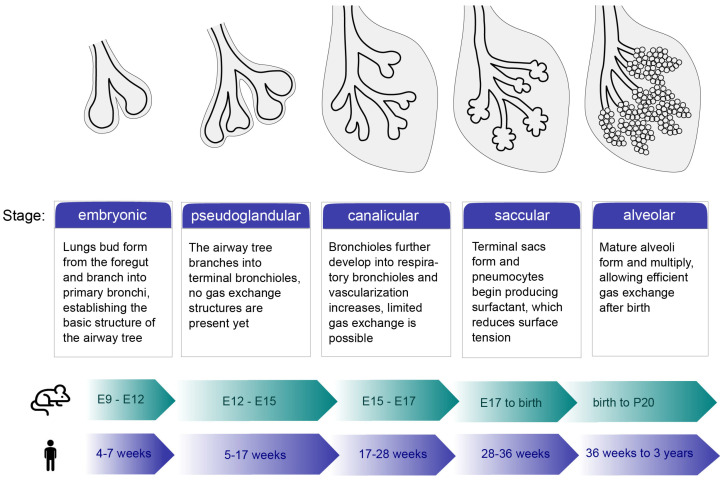
Overview of lung development stages. The process is divided into five phases: embryonic, pseudoglandular, canalicular, saccular, and alveolar. The stages of mouse development are shown in embryonic (E) days (green timeline); the stages of human development are shown in post-conception weeks (blue timeline).

The human lungs are a complex network of branched airways, blood vessels, and alveoli, forming a structure that enables gas exchange. In mammals, the pulmonary epithelium originates from the embryonic foregut endoderm [1]. In addition to epithelial cells, endothelial, mesenchymal, and immune cells are essential for the proper functioning of the lungs [2]. The development of human lungs is usually divided into five different morphological stages: embryonic, pseudoglandular, canalicular, saccular, and alveolar (Figure 1) [3]. The respiratory system begins to develop around the fourth week post-conception [4].

### 1.1. Stages of Lung Development

The embryonic stage involves the formation of lung buds at the ventral wall of the primitive foregut endoderm into the surrounding mesenchyme. At this stage, the foregut structure divides into the early trachea and esophagus [5]. The pseudoglandular stage is characterized by branching morphogenesis processes, in which the lung buds continuously divide at their tips, leading to the formation of conducting and terminal bronchioles [4,6]. After the bronchial tree is formed, the canalicular phase begins. The epithelial tips of the growing lungs begin to gradually narrow, and, as they do so, the distal epithelial progenitor cells differentiate into type I and type II alveolar epithelial cells. These cells ultimately form the future alveolar ducts [3,7].

The completion of the branching morphogenesis process marks the beginning of the saccular stage. This phase features the growth of the terminal airways, condensation of the mesenchyme, and the formation of a network of capillaries surrounding the maturing lung [8,9]. Finally, the alveolar stage involves the formation of alveoli, which increases the surface for gas exchange, and microvascular maturation. The alveolar stage begins at around 36th week post-conception and continues into early adulthood [3,10].

### 1.2. Progenitor Cells

Progenitor cells are intermediates between stem cells and fully differentiated cells. They play an essential role in organogenesis, including lung development. Dysfunction of progenitor cells can lead to congenital lung defects [8,11].

Lung epithelial progenitor cells are generally divided into two distinct stem cell subpopulations—airway and alveolar progenitors [2,9]. Airway progenitors form the lining of the tracheobronchial airways. They are organized into a pseudostratified epithelium attached to the basement membrane. In humans, the predominant types are basal, secretory (club), and ciliated cells [12]. Basal cells have the ability to self-renew and therefore constitute a population of multipotent stem cells capable of differentiating into multiple airway epithelial cell types [8,13].

Alveolar progenitors can be divided primarily into type I (AT1) and type II (AT2) alveolar cells. AT1 cells function in gas exchange, while AT2 cells secrete surfactant into the alveolus and participate in immune responses. In addition, AT2 cells constitute a population of alveolar epithelial stem cells, which, upon injury, are capable of proliferating and differentiating into AT1 cells [14,15].

### 1.3. Transcription Factors

Lung development at the molecular level is characterized by the presence of the transcription factor NK2 homeobox 1 (NKX2-1). When pulmonary progenitor cells in the foregut endoderm begin to express NKX2-1, they become committed to lung epithelial lineage fate [16,17]. Subsequently, SOX2 and SOX9 transcription factors are expressed. The expression of NKX2-1 in the endoderm is regulated by ligand molecules secreted by the mesenchyme and their signaling strictly regulates lung development by influencing the maintenance of the SOX2 and SOX9 expression gradient. The differentiated expression of SOX2 and SOX9 in epithelial cells plays a key role in their differentiation. In the proximal epithelium, progenitor cells of the developing human lung mainly express SOX2, while the epithelial cells localized in distal areas express both SOX2 and SOX9 [18,19]. Moreover, the epithelial–mesenchymal crosstalk is crucial in lung development. Signals between these two tissue types are exchanged in a way that allows them to adapt to each other as growth progresses [20].

## 2. Fibroblast Growth Factors in Lung Development

Lung growth is controlled by multiple signaling pathways involving several key ligands: fibroblast growth factors (FGFs), bone morphogenetic protein (BMP), wingless-type mouse mammary tumor virus integration site (WNT), sonic hedgehog (SHH) and vascular endothelial growth factor (VEGF) [4,21,22,23]. Among these, FGF signaling pathways constitute pivotal molecular elements mediating lung development.

### 2.1. FGF-FGFR Signaling

Fibroblast growth factors (FGFs) are a family of structurally related polypeptides with a molecular weight ranging from 17 to 34 kDa, characterized by a highly conserved core containing 120–140 amino acid residues. Their tertiary structure is characterized by a β-trefoil fold, composed of twelve anti-parallel β-strands. In addition, heparan sulfates proteoglycan (HS/HSPG) binding domains are integral parts of these proteins and play a key role in their affinity for their cognate receptors, determining the strength and efficiency of cell signaling. The FGF ligand family consists of 22 factors and is divided into seven subfamilies: (FGF1 (1/2); FGF4 (4/5/6); FGF7 (3/7/10/22); FGF8 (8/17/18); FGF9 (9/16/20); FGF15/19 (15/19/21/23), and FGF11 (11/12/13/14) [24].

**Figure 2:**
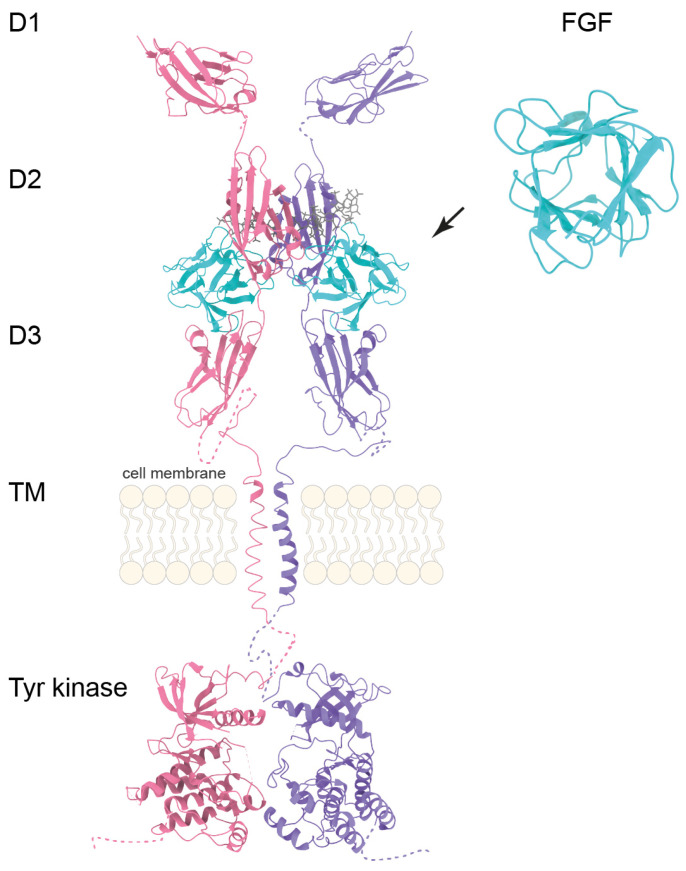
Simplified structural overview of FGF-FGFR interaction. Model of the FGFR–FGF–heparin complex based on the following structures: D1 FGFR1 (PDB:2CKN), FGFR1 (D2, D3) in complex with FGF2 and heparin (1FQ9), transmembrane domain (TM) of FGFR3 (2LZL), and the kinase domain of FGFR1 (3KY2). Color scheme: FGFR—purple and pink, FGF—turquoise, heparin—gray.

Fibroblast growth factor receptors (FGFRs) are cell surface proteins (FGFR1, FGFR2, FGFR3, and FGFR4) activated by fibroblast growth factors. FGFR1-4 consist of an extracellular region comprising three immunoglobulin-like domains (D1, D2, and D3), a transmembrane domain formed by a single helix penetrating the lipid bilayer, an intracellular region containing a tyrosine kinase domain, and an unstructured C-terminal fragment (Figure 2). FGFR1–3 undergo tissue-specific alternative splicing of the IgIII domain (D3), producing highly specific “b” isoforms in epithelial tissue and “c” isoforms in mesenchymal tissue [25].

Binding of FGF to the receptor causes receptor dimerization and leads to transphosphorylation within the tyrosine kinase domain, initiating intracellular signal transduction [26,27]. FGFRs propagate signals via PLCγ, STAT, PI3K/AKT, and MAPK pathways, controlling cell differentiation, proliferation, migration, angiogenesis, survival, and metabolism [24,28].

The diversity of FGF receptor isoforms results from alternative mRNA splicing of the D3 domain. Combined with tissue-specific expression and differential ligand binding specificity, this diversity determines the varied biological properties of individual FGFs [24]. Deregulation of this system, both at the receptor and ligand levels, contributes to a number of diseases, including cancer, developmental, and metabolic disorders [29]. Therefore, the FGF/FGFR axis remains the subject of intensive research aimed at understanding the complex interactions and their impact on physiological functions, as well as identifying potential therapeutic targets [30,31].

### 2.2. FGFs and Their Receptors in Lung Formation

During embryonic development, fibroblast growth factors mediate crucial exchange of information between cells derived from the ectoderm, mesoderm, and endoderm, guiding the growth of many organs, including the lungs. Members of the FGF family are involved in events such as early lung specification, branching morphogenesis, and lung cell differentiation and proliferation [32]. The diversity of FGF ligands and their receptors enables numerous specific ligand–receptor interactions, which further determine the fate of the cells, as well as tissues and organs.

During lung development several members of FGF family are expressed, with FGF2, FGF10 and FGF18 showing significantly higher levels. The proposed pattern of FGF expression during early lung development begins with the expression of FGF1 and FGF2, followed by FGF10, FGF8, FGF9, and FGF18. These ligands regulate each other in a cascading manner, where FGF2 induces FGF7, FGF7 in turn induces FGF1, and FGF1 helps maintain FGF7 levels. Conversely, FGF10 is likely regulated by FGF9 [33,34]. Furthermore, specific FGF receptors, in various splicing variants, are present in substantial amounts in the cells of the growing lungs, jointly highlighting the importance of FGF signaling in this process [35,36].

### 2.3. FGFRs Expression in Lung Development

Throughout the progress of lung development, all FGF receptors are expressed and operative. FGFR1 and FGFR2 are expressed in both epithelial and mesenchymal cells. It is believed that their expression pattern may influence the epithelial–mesenchymal lung branching events [36]. Yuan et al. showed that FGFR1 expression in mouse embryos shifts towards the mesenchymal compartments around embryonic day 18.5 (E18.5). At around this time, newly expressed FGFR1 accumulates in the mesenchymal AT2 niche, vascular smooth muscle cells, and cartilage of the developing trachea, rather than in any other cell populations [37]. In the pseudoglandular stage of lung development, FGFR2b expression promotes airway branching [38].

FGFR3 and FGFR4 are pivotal during lung alveologenesis [39]. Studies conducted between 11 and 18 weeks of gestation link FGFR3 mainly to epithelial cells, as its expression has been observed in the distal and proximal epithelium. In contrast, FGFR4 is found in the mesenchyme and distal epithelium [35]. Experiments focusing on the role of FGFR3 and FGFR4 during alveolar formation have shown that both receptors appear to have a cooperative function in this process. In mouse studies, combined FGFR3 and FGFR4 deficiency causes airspace enlargement and lung hypoplasia [40]. Inactivation of FGFR3 and FGFR4 in the mesenchyme disrupts postnatal elastin organization during alveologenesis. Abnormal distribution of elastin fibers affects the alveoli, which become simplified and underdeveloped [39].

### 2.4. FGF1 and FGF2

FGF1 and FGF2, members of the FGF1 subfamily, are involved in lung development by participating in the specification of the lung domain, acting in the ventral foregut endoderm [41]. FGF1 is important in early lung development. Immunohistochemical studies have confirmed its presence in lung mesenchyme [37]. This protein is also responsible for inducing epithelial budding [42]. FGF2 is active in a wide range of mesoderm- and ectoderm-derived cells [43]. The treatment of mesenchymal cells from mouse embryonic lungs with both FGF1 and FGF2 induces a concentration-dependent increase in FGF7 mRNA levels. FGF2 acts as a proliferation factor for lung epithelium and maintains FGF7 expression during development [33].

In the rat lung, FGF2 localizes to airway epithelial cells, basement membranes, and extracellular matrix (ECM). At embryonic day 13, FGF2 is found in the epithelial lining and in the basement membrane of the airway epithelium, as well as in mesenchymal and mesothelial cells and the ECM. It assists in the formation of primary bronchial buds and in determining the fate of airway epithelial cells. At E15, FGF2 is present in airway epithelial cells, with a homogeneous and diffuse presence in the ECM. At E17, it is also present in bronchial epithelium and distal airways, as well as in the extracellular matrix and smooth muscle cells around large vessels. Han et al. suggest that the dynamic expression of FGF2 in lung epithelial cells indicates its role in bronchial morphogenesis and cell proliferation, promoting branching morphogenesis. At E19, FGF2 is located at the periphery of the developing lung, correlating with intense angiogenesis and structural remodeling requiring dynamic regulation of proliferation and vasculogenesis. At embryonic day 20, FGF2 is also found in the apical membrane of the bronchial epithelium and distal airways, and at E22 it is present both intracellularly and extracellularly [44].

Overall, FGF1 and FGF2 support early lung development and mesenchymal–epithelial interactions with FGF2 acting primarily as a mitogen for the respiratory epithelium [44], which also sustains the expression of other FGF family genes during lung development [33].

### 2.5. FGF7 and FGF10

Members of the FGF7 subfamily, namely FGF7 and FGF10 (also known as KGF-1 and KGF-2, respectively) signal primarily via FGFR2b. FGF7 is expressed in both epithelium and mesenchyme during lung growth [18]. The presence of FGF7 and FGFR2b transcripts has been confirmed in rat lungs at embryonic stages E14 and E16. FGF7-FGFR2b signaling plays a role in epithelial branching. FGF7 also influences endothelial cells and acts as a crucial factor in early alveologenesis, which is mediated by secondary crest formation [45,46].

During in vitro culture of human tip epithelial progenitor cells, FGF7 induces the MAPK/ERK and PI3K/AKT pathways, which regulate proliferation by promoting columnar cell shape and maintaining junctional organization [47]. This leads to the formation of cyst-like structures that consist of undifferentiated columnar epithelial cells [48]. Despite abundant evidence for a role of FGF7 in lung development, *fgf7* knockout mice do not show lung abnormalities, suggesting compensatory role of other members of the FGF family [49].

**Figure 3:**
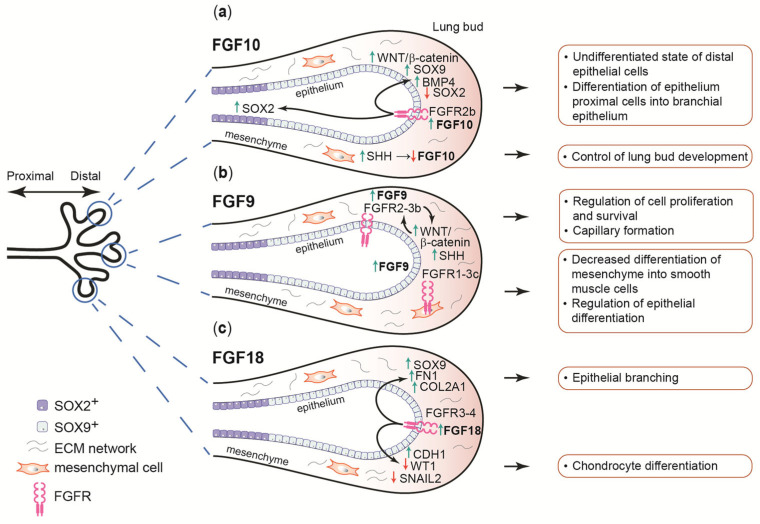
Role of selected FGFs, FGF10, FGF9, and FGF18, during lung development. (**a**) FGF10 activates the WNT/β-catenin signaling pathway by binding to the FGFR2b, which increases the expression of BMP4 and SOX9 in distal epithelial cell progenitors. FGF10 signaling also activates SOX2 expression at the proximal position. It is negatively regulated by SHH expression to precisely control lung bud development. (**b**) FGF9 indirectly participates in the regulation of WNT/β-catenin signaling by binding to FGFR2b in the epithelium. FGF9 expression is also correlated with the expression of SHH. Binding of FGF9 to FGFR1c, FGFR2c, and FGFR3c expressed in the mesenchyme results in the regulation of its differentiation into smooth muscle cells. (**c**) Binding of FGF18 to FGFR3 and FGFR4 in the mesenchyme results in increased expression of SOX9, FN1, and COL2A1, transcription factors important for the formation of chondrocytes. FGF18 signaling increases CDH1 levels and decreases WT1 and SNAIL2 levels, suggesting involvement in epithelial branching processes. Green arrows indicate increased protein expression, while red arrows indicate downregulation.

FGF10 signaling is considered a master regulator of lung development in mice and humans [50]. This protein is involved in a wide range of processes, from branching morphogenesis and control of proximal–distal patterning to epithelial cell migration and differentiation [50,51]. During lung growth, FGF10 is expressed mainly in the mesenchyme, in both distal and proximal areas (Figure 3a). The expression of its receptor, FGFR2b, was found in distal epithelial progenitors, cartilage progenitors, and airway smooth muscle cells [35,52].

Transient inhibition of FGF10 signaling in mouse embryos at E11 caused lung growth arrest—lung lobes were observed to be critically underdeveloped with impaired branching [16]. Other experiments using *fgf10* knockout and *fgfr2b* knockout mice investigated cell survival patterns. Both phenotypes were characterized by increased epithelial cell death [53] and complete lung agenesis [54,55]. Application of a stable variant of the FGF10 protein in mouse lung explants, characterized by prolonged activity relative to the wild-type variant, resulted in the inhibition of pulmonary branching and significant epithelialization. This was due to increased proliferation and differentiation of distal epithelial cells forming cyst-like structures.

Unlike TGFβ/BMP pathway morphogens, FGF protein activity is not controlled by external antagonists, indicating a different mode of signal regulation. The physiological instability of FGFs provides a mechanism that limits their action to the immediate microenvironment, where stabilization occurs through binding to surface proteoglycans. This mode of local action prevents ectopic activation of the signaling pathway in distant cells. Thus, the precise maintenance of appropriate levels of FGF proteins at different developmental stages is crucial for normal lung organogenesis [56].

The regulation of proximal–distal differentiation is driven by mesenchymal FGF10 signal mediated by FGFR2b localized on epithelial cells. This ligand, through the activation of β-catenin signaling, negatively affects the expression of the transcription factor SOX2 in distal epithelial progenitors [18,56]. This in turn induces the production of SOX9 and BMP4, which maintain the distal epithelial cell lineage in an undifferentiated state [52,57]. As the lung epithelium grows, progenitors located in more proximal positions begin to express SOX2 and differentiate into bronchial epithelium [58]. FGF10 signaling is limited by sonic hedgehog (SHH) expression in the distal mesenchyme, resulting in the inhibition of lung bud development [57,59]. Furthermore, it has been observed that FGF10 is involved in the acquisition of the basal cell differentiation fate by airway epithelial progenitors [56,57].

### 2.6. FGF9

The expression of FGF9 protein begins at the pseudoglandular stage and is observed in several locations in the developing lungs: mesothelium, distal epithelial buds, and proximal epithelial cells [35,60]. FGF9 signaling is mediated by the mesenchymal FGFR1c, FGFR2c, and FGFR3c receptor isoforms [61,62]. Mesenchymal proliferation induced by the activation of the aforementioned receptors results in increased cellular expansion and decreased differentiation of pulmonary mesenchyme into smooth muscle cells. Stimulation of the FGFR2b in the epithelium has no effect on differentiation, despite regulating canonical WNT signaling through the upregulation of *dkk1* [51,61,63].

The lungs of *fgf9* knockout mice were characterized by reduced airway branching despite preserved pneumocyte differentiation and formation of distal airspaces. This indicates that FGF9 regulates lung development mainly by stimulating mesenchyme proliferation and controlling epithelial branching [64]. FGF9 activity is also correlated with SHH signaling. SHH-maintained signal transduction from epithelium to mesenchyme controls cell proliferation and survival, as well as the expression of mesenchymal factors affecting epithelial cells (Figure 3b) [65]. The FGF9 and SHH signaling pathways also lead to the inhibition of smooth muscle cell differentiation, thus promoting their further development. Moreover, FGF9 and SHH promote capillary formation [64,66]. FGF9 also promotes epithelial fate specification, but inhibits epithelial differentiation. Research conducted by Yin and Ornitz showed that overexpression of FGF9 in mice resulted in an increase in the number of SOX9-expressing epithelial progenitors that did not produce surfactant protein C, a marker of differentiation. These data indicate that FGF9 ligand can promote epithelial fate specification but inhibits epithelial differentiation [51].

### 2.7. FGF8 and FGF18

FGF8 is involved in all steps of fetal lung development, from embryonic to alveolar stages. It is an important regulator of proliferation in lung epithelial cells. *Fgf8* hypomorphs and *Fgf8;Isl1Cre* mutants have hyperplastic lungs and exhibit increased epithelial and mesenchymal proliferation. FGF8 variant-induced intense cell proliferation at E16.5–18.5 resulted in abnormalities in the development of the epithelium, septum, and blood vessels. Importantly, when FGF8 activity is lost, its function cannot be compensated by other FGFs [62]. Mice deficient in *fgf8* die shortly after birth due to abnormalities in the respiratory system [67].

FGF18, another member of the FGF8 subfamily, is crucial for alveolar development. During alveolar secondary septation, increased FGF18 expression affects genes related to angiogenesis, cell migration and extracellular matrix production (Figure 3c). A significant increase in FGF18 expression level is observed at the beginning of alveolar secondary septation, when the protein affects the expression of elastin, a key molecule at this stage [68]. Binding of FGF18 to FGFR3 and FGFR4 can increase tropoelastin and α-SMA (smooth muscle actin) levels, suggesting a link to myofibroblast differentiation [69]. Changes in elastin expression due to FGF18 signaling have also been observed in the postnatal period [70]. FGF18 plays a key role in mouse lung morphogenesis between E11 and E14, regulating the formation of blood vessels, cartilage, and airways in the proximal direction [71]. Reduced levels of FGF18 or its receptors, FGFR3 and FGFR4, which are observed in hyperoxia or congenital diaphragmatic hernia, cause abnormalities in lung development associated with abnormal elastin deposition [72,73]. The activity of FGF18 in embryonic development is crucial during the terminal saccular phase, in contrast to FGF9 and FGF10, which are required earlier in the pseudogastric phase. At embryonic day 18.5, mice with *fgf18* deletion exhibit abnormalities in alveolar spaces, capillaries, and mesenchymal interstitial compartments [74]. In humans, FGF18 modulates lung morphogenesis at a late pseudoglandular stage of development at 11–14 weeks of gestation. FGF18 activity increases expression levels of SOX9, fibronectin (FN1), and collagen type II alpha 1 chain (COL2A1) in the mesenchyme, affecting chondrocytes differentiation in the developing airways. FGF18 also increases CDH1 levels and decreases the amount of WT1 and SNAIL, proteins responsible for epithelial branching and cell adhesion, thereby regulating epithelial branching processes [75].

## 3. The Role of FGF-FGFR Signaling in the Regeneration of Lungs

Lung repair involves complex mechanisms requiring cellular reorganization and transformation. Processes such as proliferation, migration, remodeling, and differentiation occur in a controlled sequence to regenerate lung tissue. Lung regeneration is primarily mediated by resident epithelial progenitor cells, including basal, club, AT2, and bronchioalveolar cells located in the airways and alveoli. FGF signaling pathways contribute significantly to the regulation of the occurring processes [76]. Studies have shown the important role of FGF10, along with FGF2 and FGF7, in mediating the regeneration of adult lungs [41,52].

### 3.1. Adult Stem Cell Niche Maintenance

There are several types of stem cell populations in the lungs of an adult human, which are crucial for both homeostasis and regeneration processes. Tissue damage triggers the reorganization and redifferentiation of cells residing in region-specific niches in order to restore normal lung function [4]. In the airway epithelium, basal cells appear to be the most important progenitor cells. These cells have the ability to self-renew, forming a population of multipotent stem cells that are capable of differentiating into multiple cell types in the airway epithelium [10,13]. During alveolar repair, it is mainly AT2 cells that form the alveolar epithelial stem cells niche, enabling proliferation and differentiation into AT1 cells after injury [14,15]. A brief summary of additional examples of lung progenitor cell populations is presented in Table 1.

### 3.2. Basal Stem Cells Maintenance

In the airway epithelium, the steady-state of the basal stem cell population is maintained by signaling mediated by FGFR1 and FGFR2 [87]. Experiments involving the deletion of *fgfr1* gene in tracheal basal cells in adult mice demonstrated that this receptor is essential for maintaining the quiescence of these cells in the airway epithelium of the lungs. It was found that the signal axis involved was the FGFR1-SPRY2 interaction. When this interaction was disrupted, an increase in basal cell proliferation was observed [88]. In contrast, FGFR2 signaling promotes asymmetric self-renewal of basal cells in the adult airways. FGF7 and FGF10 have been shown to be responsible for promoting self-renewal and an increase in basal cell colony size [89]. Another study indicated that during homeostasis, a localized mesenchymal stromal niche expressing FGF10 is formed in the cartilaginous airways, in close proximity to tracheal basal stem cells [38]. FGF10 signaling from the stromal tissue is involved in mechanisms of maintenance and amplification of basal progenitor cell population [52,90].

### 3.3. Alveolar Stem Cells Maintenance

FGF10-FGFR2b signaling is critical for the survival of alveolar type II stem cells in the mature lung parenchyma. Each AT2 stem cell niche is located in close proximity to a lipofibroblast that expresses FGF10 protein. Results based on *fgfr2b* inactivation showed a significant reduction in stem cell numbers, confirming that active signaling via the FGF10-FGFR2b pathway is essential for their survival and homeostasis [91]. *Fgfr2* expression is one of the factors involved in maintaining the phenotypic stability of AT2 cells by ensuring a balance between the proliferation and differentiation processes of the AT2 cells population [92]. Furthermore, deletion of *fgfr2* in alveolar epithelial type II cells resulted in a reduced AT2 cells pool, followed by alveolar enlargement and mild fibrosis [93].

### 3.4. FGFR Signaling in the Regeneration of Mature Alveoli

The mechanisms of alveolar repair in adult lung primarily involve the proliferation of alveolar type II cell population and their differentiation into alveolar type I cells, which are crucial for gas exchange processes [14]. However, recent studies suggest that the differentiation of these two types of alveolar cells may be bidirectional, as evidenced by the reprogramming of AT1 cells into AT2 cells [94].

The regeneration of mature alveoli is partly driven by FGF signaling, with pathways mediated by FGFR2 playing a significant role in the repair processes of alveoli. The alveolar epithelial progenitor (AEP) cell lineage is essential for lung tissue regeneration after injury. Under homeostatic conditions, AEP cells remain stable; however, they rapidly proliferate upon lung injury, regenerating a significant portion of the alveolar epithelium. AEP cells exhibit a distinct transcriptomic and epigenomic profile, responding to WNT, FGF7, and FGF10 signaling pathways. Studies on the function of a subpopulation of AT2 cells have shown that the lineage responds to FGFR2 activation, which promotes their proliferation. Treatment with FGF7 and FGF10 ligands has a positive effect on this process. The rapid response of AEP cells translates into the regeneration of functional alveoli [86].

The role of the FGFR2 was further investigated in AT2 cells after acute influenza infection in adult knockout *fgfr2* mice. FGFR2 was found to be involved in AT2 cell proliferation and AT2 cell differentiation into AT1 cells following lung injury, in addition to other factors such as inflammatory cytokines [92]. Dorry et al. investigated the AT2 cell population with triple conditional knockout of *fgfr1*, *fgfr2* and *fgfr3* genes in mice after bleomycin-inducted lung injury. The triple receptor deletion led to a reduction in the number of these cells, the disruption of alveolar structure, increased collagen accumulation, and increased mortality after bleomycin exposure. Subsequently, single-knockout analyses showed that FGFR2 inactivation was responsible for increased lung injury, fibrosis, and mortality, highlighting its key role in the protection and regeneration of AT2 cell [93].

### 3.5. FGF10-Dependent Regeneration of Adult Airways of the Lungs

FGF10, secreted by the distal mesenchyme, is essential for the maintenance and proliferation of distal epithelial precursors during lung development. Parabronchial smooth muscle cell progenitors (PSMCs) in this type of mesenchyme are the main source of FGF10, whose expression depends on β-catenin signaling. After epithelial damage, surviving bronchioalveolar stem cells (BASCs) begin to express WNT7b, which appears to induce parabronchial smooth muscle cells to secrete FGF10 [95,96]. In turn, FGF10 accelerates upper airway repair processes by activating BASCs, which amplify to promote the repair of damaged tissue. Transdifferentiation of BASCs into goblet cells has also been observed [95]. FGF10 has been proven to mediate the ability of the stromal niche to support epithelial regeneration and has been shown to be partially regulated by TGF-β [97]. A similar mechanism of epithelial–mesenchymal crosstalk has also been reported in the repair of cartilaginous airways. In response to the FGF10 signaling from the airway smooth muscle cell niche, basal stem cells expand and are recruited to the site of injury. The Hippo pathway, as well as WNT7b, have been shown to play a role in this process [90].

The role of FGF10 in lung protection mechanisms also includes its signaling to mesenchymal stem cells. Direct delivery of FGF10 to the lungs of rats resulted in the mobilization of the mesenchymal stem cell niche and their subsequent proliferation [98]. Another role of FGF signaling in pulmonary regeneration appears to involve the interplay between FGF10 and inflammation during lung injury [99]. In models of lung injury induced by exposure to particulate matter, FGF10 has been shown to act as an anti-inflammatory and cytoprotective agent. The mechanism of action of FGF10 is based on the suppression of the HMGB1-TLR4 signaling pathway, which results in reduced expression of pro-inflammatory cytokines (IL-6, IL-8, TNF-α) and improved lung tissue morphology [100].

Experiments investigating FGF10/FGFR2b signaling after bleomycin injury have shown that airway basal stem cells may also contribute to alveolar repair. It has been observed that overexpression of the FGF10 ligand in the bronchial epithelium can lead to differentiation of BSCs into AT2 cells. After cessation of FGF10 induction, several AT2 cells differentiated into AT1 cells [91].

## 4. Lung Dysfunctions and the Potential of FGF-Related Treatments

**Figure 4:**
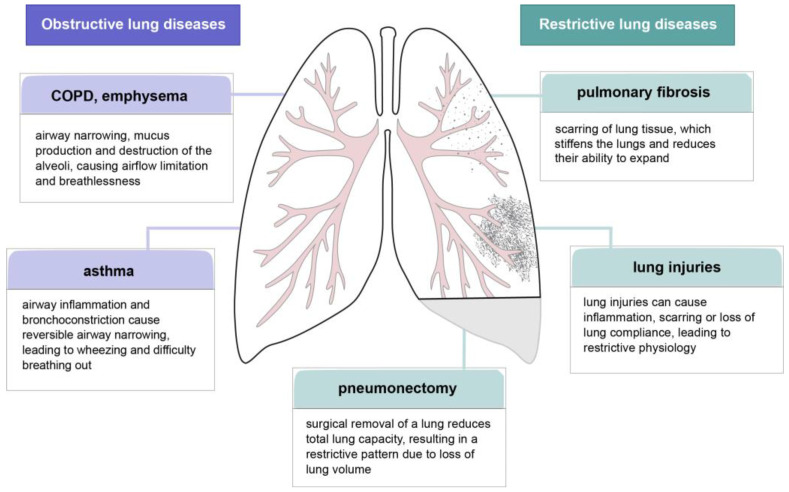
Overview of lung disfunctions. Lung conditions can be divided into two groups: obstructive lung diseases, which include chronic obstructive pulmonary disease (COPD), emphysema, and asthma, and restrictive lung diseases, which comprise pulmonary fibrosis, acute lung injuries (ALI), and pneumonectomy.

Pulmonary dysfunction is a complex condition that involves abnormal gas exchange and structural damage to lung tissues, leading to a reduction in their ability to ventilate and oxygenate properly (Figure 4). One of the underlying pathological mechanisms is the dysregulation of cell growth and tissue repair, processes strongly associated with the activity of fibroblast growth factors. FGFs, in particular FGF7 and FGF10, have demonstrated potential to regenerate damaged lung tissues by stimulating epithelial cell proliferation and improving stem cell function [38,97,101,102,103]. Research into the role of FGFs in lung repair suggests promising therapeutic possibilities for the use of these proteins to treat chronic lung diseases such as emphysema and idiopathic pulmonary fibrosis [52,104,105,106]. Therapies targeting the modulation of FGF pathways may improve lung function and slow disease progression, though further clinical trials are needed to determine their efficacy and safety [106].

### 4.1. Pulmonary Fibrosis

Pulmonary fibrosis is characterized by progressive scarring of the lungs and the resulting deterioration of lung function associated with tissue remodeling and abnormal deposition of extracellular matrix proteins. FGF signaling is implicated in pathologies leading to lung fibrosis, but the mechanisms and roles of these pathways in this process have not been fully elucidated [107]. Treatments targeting FGF-dependent signaling are still being developed, and some are undergoing clinical trials [108]. Inhibition of FGFRs offers a potential therapeutic approach for pulmonary fibrosis. One of the drugs currently in use is the receptor tyrosine kinase inhibitor, Nintedanib (BIBF1120), which blocks FGFR-FGF signaling by binding to FGFR and reduces lung fibrosis [109]. Preclinical studies have also confirmed the efficient effect of the ZSP1603 tyrosine kinase inhibitor, which specifically targets FGFR1-4. In animal models of bleomycin-induced pulmonary fibrosis, the use of the inhibitor has been shown to reduce lung damage compared to the control group and also to impede the differentiation of fibroblasts into myoblasts [110,111]. Another inhibitor of the FGF-FGFR signaling pathway is the soluble ectodomain of fibroblast growth factor receptor 2 IIIc with the S252W mutation (msFGFR2). Yu et al. demonstrated that binding of msFGFR2 to FGFR2c blocks signaling pathways that play a key role in the pathogenesis of pulmonary fibrosis. Treatment with msFGFR2 reduced the expression levels of α-SMA and collagen in mice, alleviating the symptoms of pulmonary fibrosis [112].

FGF proteins are also involved in the regeneration of airway epithelial cells after damage caused by pulmonary fibrosis. Inactivation of FGFR2b in bronchial epithelial stem cells results in impaired regeneration of the alveolar epithelium. The expression levels of the ligands of FGFR2b, FGF10 and FG7, are lower in fibrotic lungs undergoing active remodeling [113]. Overexpression of FGF10 and subsequent activation of FGFR2b signaling promotes regression of idiopathic lung fibrosis by inducing the differentiation of bronchial epithelial stem cells into alveolar epithelial cells [108]. It is worth noting that the stable variant of FGF10 exhibits regenerative potential in ex vivo models with early fibrotic changes. Treatment of fibrotic lung fragments with stable FGF10 resulted in a reduction in lactate dehydrogenase secretion and a decrease in the level of α-SMA positive cells [56].

Unlike members of the FGF10 family, whose cognate receptor is FGFR2b, FGF2 signals primarily through FGFR2c [93,114]. The expression of this ligand in the alveolar epithelium after bleomycin-induced damage has been observed in murine lungs and human idiopathic pulmonary fibrosis explant lungs. The FGF2 ligand has been shown to contribute to the regeneration of mature alveolar epithelium after injury. In *fgf2^−/−^* mice, increased mortality and impaired epithelium barrier recovery were observed compared with to the control group [115]. Interestingly, in contrast, when FGF2 is induced in response to TGF-β1 treatment, it induces activator protein-1, which is critical for the activation of genes involved in pulmonary fibrosis [116], and also increases α-SMA, enhancing this process. The use of FGFR2c ectodomain (sFGFR2c) as an FGF2 antagonist in a mouse model has been shown to be useful in the treatment of pulmonary fibrosis [117]. In addition, other FGF proteins, FGF1 and FGF9, reduce the levels of extracellular matrix proteins (collagen-1 and α-SMA), suggesting their role in inhibiting the differentiation of fibroblasts into myoblasts in the pathogenesis of the disease [118].

Endocrine FGFs, FGF19, FGF21, and FGF23, may also be beneficial in the treatment of pulmonary fibrosis. In a mouse model, overexpression of FGF19 attenuated fibrotic changes induced by bleomycin or TGF-β injection, suggesting the anti-fibrotic properties of FGF19 [119]. Oxidative stress is also one of the causes of pulmonary fibrosis, leading to the formation of TGF-β, which in turn affects the deposition of extracellular matrix proteins, thus promoting fibrosis processes. It has been shown that the anti-fibrotic effect of FGF21 may be related to its antioxidant activity and involves the activation of Nrf-2 signaling along with a reduction in TGF-β expression [120]. The pro-fibrotic effect of TGF-β is also compensated for by the activity of FGF23 and the β-Klotho protein [121].

### 4.2. Lung Injuries

Acute lung injury (ALI) is a serious condition characterized by lung inflammation and loss of epithelial integrity, leading to fluid accumulation in the alveoli, which can be caused by a variety of factors, including infection, trauma, or inhalation of harmful substances. Through their involvement in epithelium reconstruction of the respiratory airways, fibroblast growth factors and their signaling are strongly implicated in ALI-related pathologies.

Viral infections represent a substantial subset of respiratory diseases. One of the most common is influenza virus infection, characterized by high morbidity, which can lead to respiratory failure caused by alveolar edema [122]. Increased expression of FGF2 has been observed in patients infected with influenza A virus (IAV). In a mouse model, administration of recombinant FGF2 has been shown to alleviate the effects of lung injuries. Through NF-κB activation, FGF2 affects neutrophil recruitment, which is important in the repair processes of ALI caused by influenza virus H1N1, a subtype of IAV [123]. FGF9 also plays a crucial role in IAV pathology, but through a different mechanism. Club cell-derived FGF9 signaling may directly disrupt the integrity of the airway epithelial barrier, allowing the virus to enter the alveolar space at an early stage of infection, or alternatively, exert a paracrine effect on alveolar epithelial cells, increasing their susceptibility to infection. In an in vivo study, mice overexpressing FGF9 showed high levels of type I interferon and pro-inflammatory cytokines in the conducting epithelium, resulting in the development of inflammation and subsequent lung damage [124].

FGF7 treatment has been explored for enhancing alveolar injury resistance. In mice with inducible FGF7 expression, inhibition of hyperoxia-induced lung epithelial cell death was observed [125]. Increased expression of aquaporin 5 in vivo after FGF7 stimulation [126] and the involvement of FGF7 in the regulation of epithelial sodium channels (sodium potassium pump Na^+^/K^+^ ATPase) and epithelial chloride channel Clc-2 contributed to the removal of excess fluid from the lungs during edema [127,128], supporting the process of lung repair [129]. However, research carried out by Nikolaidis et al. has shown the opposite effect. Intratracheal administration of FGF7 increased mortality and exacerbated IAV symptoms in a mouse model due to the increased susceptibility of type II alveolar cells to virus infection under the activation of the PI3K/AKT/mTOR signaling pathway [124].

FGF18 affects mesenchymal progenitor cells, promoting chondrogenic differentiation and modulating epithelial–mesenchymal interactions, which are essential for lung branching morphogenesis and regeneration [76]. Treatment of endothelial cells with FGF18 has been shown to significantly reduce LPS-induced expression of VCAM-1, ICAM-1, IL-6, and TNF-α, proteins that are biomarkers of pulmonary endothelial injury. Therefore, FGF18-based therapy may be a promising therapeutic strategy for the treatment of acute lung injury. Studies have shown that FGF18 inhibits the phosphorylation of IκBα and NF-κB p65 in a mouse model of ALI and reduces nuclear accumulation of NF-κB p65, resulting in attenuated cellular inflammation and accelerated lung repair [130].

### 4.3. COVID-19 Infections

FGF/FGFR signaling may also be a target in the treatment of lung inflammation associated with COVID-19 infections. Calcium Dobesilate, an inhibitor targeting FGF-regulated pathways, blocks the transcription of TMPRSS2, a protein essential for the entry of SARS-CoV-2 virus into lung cells. In addition, Calcium Dobesilate prevents the virus from binding to heparan sulfate on the surface of endothelial cells, reducing the virus’s ability to infect [131,132,133]. In lung tissue samples collected after death due to COVID-19, changes in blood vessel morphology and the presence of new vessels were observed, which correlated with changes in FGF2 expression [134]. FGF2 is known as a pro-angiogenic factor and can therefore be considered as a marker in diseases associated with pulmonary fibrosis, including COVID-19 [135]. Patients with moderate disease have elevated FGF2 levels, while critically ill patients have reduced FGF2 expression. Thus, it can be concluded that FGF2 is involved in the regulation of COVID-19 development and may be a potential therapeutic agent [136]. Similarly, FGF21 expression levels are correlated with disease progression. During recovery from COVID-19, FGF21 levels increase significantly, so monitoring them may help track the healing process [137].

### 4.4. COPD and Emphysema

Chronic obstructive pulmonary disease (COPD) is a progressive, life-threatening clinical syndrome involving chronic bronchitis and structural changes in the lungs. The disease is characterized by destruction of lung tissue and impaired lung repair capacity [138]. COPD is associated with damage to the endothelial glycocalyx and endothelial cell apoptosis [139]. The resulting loss of the protective functions of the glycocalyx leads to the deregulation of vascular homeostasis and vascular barrier permeability, followed by the activation of thrombosis [140,141]. Apoptosis of endothelial cell contributes to the destruction of lung tissue and the development of emphysema [142].

FGF/FGFR signaling plays an important role in the pathology of COPD. FGF10 activity, by reducing apoptosis in endothelial cells and increasing the levels of glycocalyx components (heparan sulfate, chondroitin sulfate, and syndecan-1), leads to the repair of glycocalyx damage and effectively alleviates COPD symptoms [138]. Upregulation of FGF1, FGF2, and FGFR1 expression has been demonstrated in the bronchial epithelium and smooth muscles of airways, which may suggest a role for these molecules in airway remodeling [143]. In COPD patients exposed to cigarette smoke, increased expression of FGF23 was observed, correlated with reduced Klotho levels, which resulted in the activation of the pro-inflammatory FGFR4/PLCγ/NFAT signaling pathway. This suggests that dysregulation of FGF23 and Klotho may be used as a marker in the diagnosis and treatment of COPD [144,145].

Emphysema, a COPD-associated condition, entails significant alveolar damage and airspace enlargement. To date, no effective regenerative therapies have been developed [146]. FGF10 appears to be an important potential component in the treatment of emphysema and pulmonary hypertension, as demonstrated in studies on mice exposed to cigarette smoke or elastase [106]. Studies indicate that FGF10 deficiency promoted the development of both conditions, while overexpression of this factor may lead to their successful reversal. FGF10 activity attenuates the development of emphysema and reduces the loss of glycocalyx after exposure to cigarette smoke by activating HS6ST1 (heparan sulfate 6-O sulfotransferase 1), which is a key enzyme in the biosynthesis of heparan sulfate, the main component of glycocalyx [139]. In an elastase-induced emphysema model, increased cell proliferation was observed after FGF10 stimulation. Treatment of mouse precision cut lung slices (PCLS) with a stable mutant of FGF10 increased the number of cells expressing ProSPC, a marker of AT2 cells, indicating the regenerative properties of this variant [56].

FGF2 also plays an important role in the regeneration of lungs by emphysema [147]. Administration of recombinant FGF2 alleviated the symptoms of COPD-associated emphysema in mice subjected to short-term exposure to cigarette smoke [148]. Intratracheal administration of FGF2 has also been shown to affect lung blood flow and restore lung function in a model of emphysema in dogs [149]. Furthermore, treatment of mice with early-stage emphysema with FGF2 fused to a collagen-binding domain (CBP-FGF2) reduced inflammation by lowering the levels of the pro-inflammatory cytokines IL-6 and TNF-α [146].

FGF7 has also been shown to have anti-inflammatory and protective effects against the development of elastase-induced emphysema. Mice treated with FGF7 showed lower levels of chemokines CCL2 and CXCL2, as well as ICAM-1 and VCAM-1 proteins, and reduced activity of MMP-2 and MMP-9 metalloproteinases, suggesting an effect on the development of inflammation [105]. Therapy with modified FGF7, ΔN23-KGF, also known as palifermin, has been shown to induce processes related to the maintenance of alveolar structure, preventing the progression of emphysema and leading to alveolar regeneration. The molecular and cellular mechanisms underlying this process include the stimulation of proliferation, migration, and differentiation of type II alveolar epithelial cells, effects on the extracellular matrix, particularly elastin fibers, and increased expression and activation of TGF-β1 and TGF-β2 [104]. Interestingly, single-nucleotide polymorphisms of the FGF7 gene are strongly correlated with the occurrence of COPD [150,151].

In addition to inflammation, emphysema may also result from morphogenetic defects. Abnormalities in FGF protein signaling at the pseudoglandular and canalicular stages can lead to permanent changes in lung structure. In a mouse model, it has been shown that prenatal inhibition of FGF protein distribution reduces the number of type II epithelial cells and pulmonary vascular density, leading to the development of emphysema. Proper FGF signaling during critical stages of prenatal development is essential for the formation of functional lung architecture, and its disruption can lead to irreversible changes typical of emphysema [152].

### 4.5. Pneumonectomy

Pneumonectomy (PNX), a condition resulting from the removal of part of the lung, in animals leads to compensatory growth of the remaining lung lobes. This reflects the loss of gas exchange units, as in chronic lung disease, but unlike these models, the tissue loss is well-defined, reproducible, and not associated with inflammation [153]. During lung regeneration after pneumonectomy, cell proliferation occurs throughout the lung parenchyma, involving mesenchymal, endothelial, and epithelial compartments [154]. Lung regeneration post-unilateral PNX is age-dependent; notably, lung mesenchymal stromal cell (LMSC) proliferation declines with age in mice [155]. These changes are evident both in the number of cells and their ability to proliferate after PNX. The main factor contributing to the decline in LMSC proliferation in older mice is a decrease in the expression of genes responsible for the self-renewal and differentiation of these cells. This applies in particular to genes associated with retinoic acid (RA), FGF/WNT and elastogenesis pathways. Of particular note is the reduced expression of the *fgfr1* gene under these conditions [156]. It has been suggested that FGF signaling intersects with specific pathways, including EGF, WNT, and Notch, engaged in the proliferative and tissue-maturation phases after lung resection [157,158,159].

### 4.6. Asthma

Asthma is a chronic respiratory disease with genetic and environmental causes that can occur at any age, and its triggers are poorly understood. Asthma may be caused by autoimmune factors, and the symptoms of the disease are characterized by a high degree of heterogeneity. Patients suffering from this condition experience airway remodeling, progressive inflammation, and bronchial hyperresponsiveness [160,161]. In a model of asthma induced by house dust mite, elevated FGF2 levels have been shown to correlate with increased inflammation. The pro-inflammatory action of FGF2 is mediated by activation of the FGFR/MAPK/NF-κB pathway [162]. Although inhaled corticosteroids (ICS) are one of the available treatments for asthma, some patients do not notice improvement with this therapy [163]. A link has been demonstrated between eosinophilic and steroid-induced neutrophilic inflammation caused by ICS administration and the FGF-dependent pathways. Increased expression of FGF1, FGF2, FGF4, and FGF18 in human lung tissue has been shown to increase airway inflammation in response to ICS. Future therapy targeting blockade of the FGFR signaling may help treat asthma in ICS-resistant patients [164].

## 5. Potential Therapeutic Approaches Targeting the FGF-FGFR Pathway for the Treatment of Lung Dysfunctions

FGF-FGFR signaling is crucial for proliferative, anti-apoptotic and metabolic processes, as well as maintenance of homeostasis in the body [26]. Fibroblast growth factors are involved in many processes within lung development, but their exact function has not been definitively established [3]. The numerous and diverse biological processes controlled by FGF proteins and the signaling pathways they activate indicate their significant therapeutic potential, underscoring the importance of research into their medical applications, including lung regeneration.

Importantly, both increased activation and downregulation of FGF-FGFR signaling have been shown to be associated with respiratory diseases. Different FGFs may exhibit both pro-inflammatory and pro-regenerative effects; therefore, their potential for use in the context of lung damage prevention or regenerative medicine should be comprehensively evaluated. Modulation of FGFR signaling may have positive effects in the treatment of conditions such as pulmonary fibrosis, lung injury, COPD, emphysema, pneumonectomy and asthma [29]. For example, FGF7 has shown beneficial effects in a hyperoxic bronchopulmonary dysplasia model [165], while FGF10 has exhibited protective effects in cases of pulmonary fibrosis and emphysema [106,166]. On the contrary, in cases where excessive FGF signaling is associated with lung function pathology, a number of inhibitors targeting FGFRs have been developed, including Nintedanib, ZSP1603 and Calcium Dobesilate, which alleviate pathological symptoms in fibrosis and lung injury. However, they are all non-specific inhibitors that are not directed exclusively towards FGFR activity [110,111,112,131].

Furthermore, one of the challenges in developing therapies employing FGFs is their low stability [167]. The stability of proteins is crucial for their pharmacological application. Genetic engineering techniques make it possible to precisely modulate the properties of recombinant proteins to increase their application potential. Enhancing the structural stability of FGFs using different approaches, and thus extending the lifespan of the proteins, significantly increases the protective effects in the treatment of various diseases [168]. Thus, for example, a stable variant of FGF10 shows stronger regenerative properties compared to the wild type protein in ex vivo models of lung injury [56].

The development of new therapies using FGF in lung regeneration requires careful consideration of the appropriate method of drug administration. Due to the structure of the lungs, their large surface area and permeability, inhaled preparations are often used to treat respiratory diseases. There are many advantages to using inhalers: faster, localized drug delivery, reduction in systemic effects, and elimination of gastrointestinal absorption problems [169]. Inhaled administration of recombinant FGF10 was shown to accelerate regenerative processes in lung tissues exposed to smoke in a rabbit model [170].

Importantly, due to the low stability of FGF family members, it is essential to select the appropriate formulation. The use of FGF2 in lung regeneration in the form of a dry powder for inhalation has been reported. The addition of 20% (w/w) leucine to the formulated powder allowed the biological activity of FGF2 to be maintained [171]. The administration of recombinant stable variants of FGFs by the inhaled route may be a promising strategy in the development of lung disease therapies. It is important to note that not only low levels of FGF proteins, but also excessive activation of FGFRs generated by these proteins can lead to various pathological conditions, including lung dysfunction. The FGFR inhibition approach is a significant tool already used in cancer treatment [172]. It appears that blocking of FGF signaling by inactivating FGFRs with receptor and/or isoform specific molecules, such as small molecule inhibitors, monoclonal antibodies and their fragments or aptamers, may also be utilized as a strategy aimed at treating certain lung conditions.

In addition, transcriptomic and histopathological analyses of samples from patients with lung diseases may prove useful in selecting appropriate therapy. Verification of the expression levels of genes encoding FGFs and the levels of synthesized proteins can provide information on the amount of specific ligands that can be compensated for by giving the patient appropriate drugs, such as growth factors or ligand traps. Such personalized therapy aimed at rebalancing FGF proteins to achieve homeostasis could accelerate regenerative processes.

Also, modern strategies related to gene editing are likely to lead to the development of effective treatments for lung diseases. It has been shown that the CRISPR method can be used to treat cystic fibrosis, a disease that also affects the respiratory system [173]. The CRISPR/Cas9 strategy in the context of FGF proteins has already been used successfully to treat osteoarthritis by increasing FGF18 levels [174]. These data suggest that editing of FGF-FGFR-associated genes may also find application in the regeneration and treatment of lung diseases in the future. Undoubtedly, however, the development of effective therapies requires future research to deepen our understanding of the mechanisms involved in the role of FGF signaling in lung dysfunctions.

## Figures and Tables

**Table 1:** Overview of examples of pulmonary stem cells populations.

	Cell Type	Differentiation Capability	Location	References
Airway stem cells niche	Basal stem/progenitor cells	Self Airway luminal cells	Epithelium of the tracheobronchial tree	[10,77,78]
	Club (secretory) cells	Self Ciliated cells AT2 and AT1 cells	Epithelium of the tracheobronchial tree	[79,80]
	Myoepithelial cells	Self Basal cells Luminal cells	Submucosal gland	[81]
	Distal airway cell populations	Self Basal cells Club cells Ciliated cells AT2 and AT1 cells	Distal airways regions	[82,83,84,85]
Alveolar stem cells niche	Alveolar type II cells	Self AT1 cells	Lung alveolus	[14,86]

## Data Availability

No new data were created or analyzed in this study.

## Abbreviations

The following abbreviations are used in this manuscript:

α-SMA	smooth muscle actin alpha
AEP	alveolar epithelial progenitor
ALI	acute lung injury
AT1	alveolar type I
AT2	alveolar type II
BASCs	bronchioalveolar stem cells
BMP	bone morphogenetic protein
COL2A1	collagen type II alpha 1 chain
COPD	chronic obstructive pulmonary disease
ECM	extracellular matrix
EGF	epidermal growth factor
FGF	fibroblast growth factor
FGFR	fibroblast growth factor receptor
FN1	fibronectin
IAV	influenza A virus
ICS	inhaled corticosteroids
LMSC	lung mesenchymal stromal cell
MAPK	mitogen-activated protein kinase
PI3K/AKT	phosphatidylinositol 3-kinase—protein kinase B
PLCγ	phospholipase C gamma
PNX	pneumonectomy
PSMCs	parabronchial smooth muscle cell progenitors
SHH	sonic hedgehog
STAT	signal transducer and activator of transcription
TGF-α/β	transforming growth factor alpha/beta
TM	transmembrane domain
VEGF	vascular endothelial growth factor
WNT	wingless-type mouse mammary tumor virus integration site
